# Thai Older People’s Willingness (Intention) to Participate in a Care Prevention, Community Group Exercise Program: An Assessment before Implementing an Intervention Trial in Chiang Mai, Northern Thailand

**DOI:** 10.3390/ijerph18084044

**Published:** 2021-04-12

**Authors:** Thin Nyein Nyein Aung, Myo Nyein Aung, Saiyud Moolphate, Yuka Koyanagi, Nadila Mulati, Siripen Supakankunti, Motoyuki Yuasa

**Affiliations:** 1 Department of Public Health, Graduate School of Medicine, Juntendo University, Tokyo 113-8421, Japan; a-thin@juntendo.ac.jp (T.N.N.A.); m.nadila.vp@juntendo.ac.jp (N.M.); moyuasa@juntendo.ac.jp (M.Y.); 2 Advanced Research Institute for Health Sciences, Juntendo University, Tokyo 113-8421, Japan; 3 Faculty of International Liberal Arts, Juntendo University, Tokyo 113-8421, Japan; 4 Department of Public Health, Faculty of Science and Technology, Chiang Mai Rajabhat University, Chiangmai 50300, Thailand; saiyudmoolphate@gmail.com; 5 Department of Medical and Health Science, Tokyo Ariake University, Tokyo 135-0063, Japan; koyanagiy@tau.ac.jp; 6 Centre of Excellence for Health Economics, Faculty of Economics, Chulalongkorn University, Bangkok 10330, Thailand; Siripen.S@chula.ac.th

**Keywords:** aging, community-integrated intermediary care (CIIC), exercise, functional training, health promotion, older adult, Thailand

## Abstract

Background: Sustainability of a current family-based long-term care model in Thailand has been challenged by demographic aging, and the rising burden of non-communicable diseases and age-related morbidities. In order to overcome these challenges, a community-integrated intermediary care (CIIC) service model, including functional training as one of the interventions, was introduced. To increase program uptake and adherence, it is vital to understand the facilitators and barriers for joining this group exercise. Therefore, we aimed to explore the determinants of older adults’ willingness to participate in it. Methods: A total of 1509 participants from an intervention arm of a cluster randomized trial were interviewed with a structured questionnaire. A descriptive analysis and binary logistic regression were applied. Results: More than half of participants (51.4%) were willing to join community-based care prevention, functional training exercise program. Significant associated motivators were being of younger age, female, married, working seniors, original community residents, having (regular and irregular) exercise habits, history of a fall in the last six months, needs for walking aids and secondary caregivers. Conclusion: Our findings highlighted both personal and social factors motivated old people to access health promotion activities. It might help design or implement effective programs to promote healthy aging among community-dwelling seniors in Thailand. This research is analysis of baseline results from a Community Integrated Intermediary Care project. TCTR20190412004.

## 1. Introduction

Countries all around the world are experiencing the demographic change of aging populations. Aging in Asia is the fastest in the world and Thailand ranks fourth, following Japan, South Korea, and Singapore. In 2035, 23.1% of the Thai population will be aged 65 years or more [1]. The family members are informal caregivers and a family-based long-term care system is currently being practiced in Thailand although its sustainability is being challenged by the reduction in family sizes due to the migration of children for their job opportunities [2]. Population aging, associated with an increased burden of Non-communicable diseases (NCDs), presents challenges to individuals, families, communities, and society. Deaths caused by NCDs are common worldwide and the global death rate attributed to NCDs is rapidly increasing in low- and middle-income countries. NCD mortality rates in Thailand also increased from 64% in 2000 to 73% in 2015 [3]. The health of older persons will not only affect themselves but will also have an impact on their family caregivers. Facing a rising burden of NCDs and age-related morbidities, Thai communities need health promotion programs to support and sustain community integrated care for older people [4,5]. Therefore, the community-integrated intermediary care (CIIC) service model which aims to reduce the burden of family caregivers and promote older adults’ functional ability and quality of life will be introduced to Thailand.

A socially and economically engaged, secure, and healthy aging population can bring endless opportunities to this demographic shift. Hence, there has been an increased number of researches focused on understanding and intervening to support factors that affect health and well-being of older adults. However, opportunities come with challenges. Regular physical activity has been proven to help prevent and treat NCDs [6,7]. According to the World Health Organization, physical inactivity is the fourth leading risk factor for global mortality [8]. In Thailand, the data from a 2015 population-representative survey on physical activity showed that most older persons did not reach the recommended physical activity level and had a high rate of the high level of physical inactivity [9]. As such, a challenge confronting the field of preventive medicine is to develop and implement efficacious interventions and initiatives for the older persons that can support their sustained engagement in health-enhancing physical activity. Community-based exercise interventions have been developed to improve older people, staying healthy and active for as long as possible [10]. Improvement of both physical and cognitive functions has been expected by increasing their opportunities to increase physical activity and socializing with peers. The regular group exercise helped the elderly to improve or maintain their functional health and enjoy their lives. Caring for others and supporting each other can also help elderly people feel socially connected and experience a sense of security in the community [11]. Japanese studies also noted that there was a positive effect of three months functional training on muscle strength, function, and cognitive improvements in the elderly [12,13]. Furthermore, functional training can promote the activeness and independence of senior citizens to prevent care needs [14].

Although physical activity promotion campaigns were widely implemented across Thailand, care prevention exercise program for older persons have not been systematically launched yet. Existing literature did not show the willingness of the older people in the participation of community-based exercise program for preventing long term care. There is a research gap to assess the willingness of older Thai people to participate in the community-based care prevention program. Meanwhile before launching the intervention for CIIC project, it is necessary to determine the intention of community residents, potential participants, to participate in the exercise program.

Health related behaviors can be determined by the person’s functional or health status, both their physical and mental wellbeing and social-culture or environment [15,16]. Moreover, based on Albert Bandura’s social cognitive theory, human functioning is also the outcome of the relationship between behavioral, personal, and environmental influences [17]. Consequently, a better understanding of associated factors that influence the willingness of elderly people to participate in the functional training exercise will be important when introducing an effective community group exercise program for Thai elderly, as it could increase program participation and adherence which can then open doors to the creation of an empowered community.

## 2. Materials and Methods

### 2.1. Data Collection and Participants

This study was conducted in accordance with the Declaration of Helsinki. The World Health Organization Ethical Review Committee: WHO/ERC ID; ERC.0003064, dated 7 March 2019 and Ethical Review Committee for Research in Human Subjects: Boromarajonani College of Nursing Nakhon Lampang: Praboromarajchnok, Institute for Health Workforce Development, Ministry of Public Health, Thailand (approval number E 2562/005, dated 4 March 2019) approved the ethics of the study. It has been registered at the Thailand Clinical Trial Registry, Trial registration number TCTR20190412004. It was a cluster randomized controlled trial, comprising six intervention clusters and six control clusters that aimed to recruit 2000 participants in each arm. The community area in Chiang Mai Province was chosen to be the study area as it is the most populated province in the north; 18.2 percent of the entire population are older persons [18]. The area of Maehia Subdistrict, Mueang Chiang Mai District was chosen to be the intervention arm by cluster randomization. STATA version 11SE (Stata Corporation, College Station, TX, USA) was utilized for sample and power estimations. The precision levels applied are a *p*-value of 0.05 with a 95% confidence interval.

#### Community Based Exercise for Prevention of Long-Term Care

We aimed to introduce functional training in the form of a community group exercise program to promote active and healthy ageing. A care prevention exercise program for Thai elderly will be designed and developed by a Japanese expert and delivered to Thai community dwelling elderly in the form of a community group exercise. The unique feature of this program is functional training and exercise accompanied by stretching, where both can be achieved either by standing or sitting. It consists of a total of 24 sessions (two sessions per week for 12 weeks) and each session will comprise 10 min of stretching, 30 min of functional training and exercises, followed by 10 min of cooling down and stretching. The research assistants and community volunteers will be trained by a Japanese expert and several awareness campaigns to motivate elderly people to increase access to exercise program will be done.

We conducted a pre-intervention survey. The study population in this survey is all the participants who enrolled into the intervention arm of the CIIC cluster randomized controlled trial, according to the CIIC protocol [19]. The inclusion criteria of study were defined. Inclusion criteria were persons aged 60 year and above, either male or female, residents in the study, site and voluntary participation with their written informed consent. The unconsenting people, people who can’t understand the explanation for the informed consent, and people with cognitive impairment or severe impairment in decision making abilities were excluded. The research assistants were trained for data collection and data were collected via interviewer administered survey questionnaires in 2019. Participants were recruited in six clusters which were randomly selected from the list of villages in the intervention city. The sampling frame was the list of villages in the intervention site city.

### 2.2. Measures

The structured questionnaires included the demographic characteristics of the elderly, their willingness to join with the community group exercise program and associated factors. The baseline measures were organized by different domains approximating the sociodemographic and biological, personal and behavioral, sociocultural and physical environment determinants. Socio demographic and biological determinants included age, gender, education, marital status, currently working or not. Personal and behavioral determinants which included physical health status by assessing Barthel’s activity of daily living (ADL) scores, having underlying diseases, history of a fall during the preceding six-month period, functional impairment (the need for walking aids/equipment), smoking, drinking, exercise habit, and overall wellbeing by assessing self-perceived health status rating. Social determinants were assessed by living arrangement, social support by significant others, having primary and secondary caregivers or not. The determinant of physical environment was by residential neighborhood whether older adults was living in the original village or private housing estate.

The outcome of this analysis was a single-item question whether a participant enrolled to the intervention trial want to join the community-based exercise program for the older adult. It was a yes/no question. Given that the participants were older adults and being not familiar to complex psychometric questions, we chose this approach. Moreover, we also asked whether elderly participants exercise or not, using a simple question, “Do you exercise?” to be answered in a single best response among “No”, “Yes but not regularly”, “Yes regularly” (“Exercise regularly” means “minimum 30 min of exercise per day, at least 5 days a week”).

Besides, we used an internationally validated instrument, “Barthel Activities of Daily Living (ADL) Index“ which was carefully translated with a standard procedure of translation, back translation, cognitive test and pilot study according to the WHO process of translation and adaptation of instrument [20]. It has been validated internationally [21] and commonly used for screening in Thailand [22]. We also conducted a pilot study in Chiang Mai province to determine comprehensibility, age-friendliness and readability of the questionnaires before data collection. Reliability coefficient Cronbach alpha of Thai version ADL was 0.9 for current study population. Data was collected by well-trained research assistants, interviewers.

### 2.3. Data Analysis

We used IBM SPSS version 22 (IBM Corporation, Armonk, NY, USA) for data analysis. Data were cleaned, recoding the variables and computing the subscales and scales as needed and an exploratory analysis was conducted. Demographic characteristics and socioeconomic status variables were analyzed by a descriptive analysis. Frequency and percentage were used for the categorical variables, stratified by their willingness to participate in a community group exercise and the mean (M) and standard deviation (SD) were used for continuous variable (age). Binary logistic regression was applied to determine the factors associated with their intention to join the community group exercise program. An adjusted odds ratio (adj OR) with a 95% confidence interval and a *p* value of <0.05 were considered to be significantly associated factors.

## 3. Results

### 3.1. Sample Characteristics

**Figure 1:**
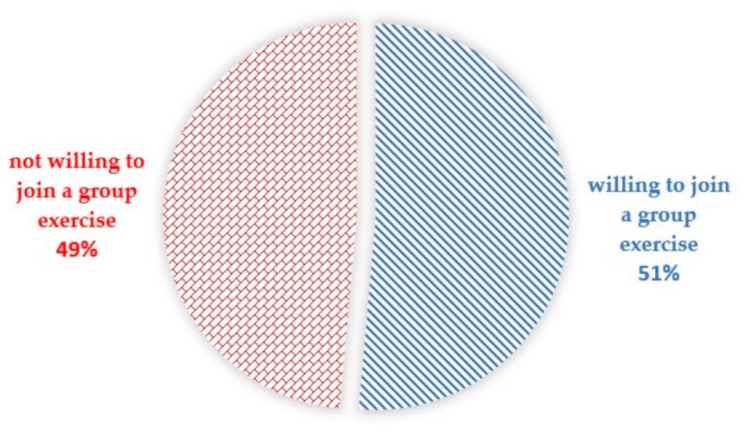
Willingness (intention) of Thai older persons to participate in a community group exercise, Maehia, Chiang Mai, Thailand 2019.

The results were from the baseline survey of an intervention arm. A total of 1509 older adults, with a mean age of 69.31 ± 7.10 years, were included in this study. About 61.8% of the study participants were females and the majority (80.8%) were original community residents. Table 1 provides the socio-demographic characteristics of the respondents. More than half of the older adults were married (59.8%) and the spouses contributed 35.5% of the primary family caregiver, following son/daughter or son/daughter in law (40.6%), and 90.1% of the seniors had primary caregivers and 53.2% had secondary caregivers. In regards to the living arrangement, 13.1% of the study participants were living alone. About 5% of the study participants did not have any formal education and 43.8% completed primary school. Working older adults accounted for 30.9% of the respondents and only 2.3% had moderate to severe dependency in terms of Barthel’s ADL total scores. Regarding physical health, 950 of them (63.0%) had underlying diseases, and 156 of them (10.3%) experienced falls in the past six months. Nearly half of them perceived that their overall health status was good/very good (48.2%). About 266 older adults (17.6%) were in need for walking aids or equipment. In terms of lifestyle behaviors, current smokers and alcohol drinkers made up 6.5% and 19.5% of the sample respectively. The majority of the older adults (70.8%) did not have regular exercise habit. When asked about the willingness to engage in a functional training community group exercise, more than half of the older adults (51.4%) answered yes (Figure 1).

### 3.2. Willingness to Participate in a Community Group Exercise and Associated Factors

Binary logistic regression analysis indicated statistically significant associations (*p*-value ≤ 0.05) between the elderly’s willingness to join a community group exercise and several factors, and the results are given in Table 2. Study participants in the younger age group (less than 75 years old) were more likely to participate in the community group exercise program compared to the participants in the older age group (greater than 75 years old) (Adjusted odd ratio (Adj OR) = 1.39, 95%CI: 1.05, 1.85). Female participants were more likely to join the functional training program compare to the male participants (Adj OR = 1.69, 95%CI: 1.33, 2.15). Seniors who were living in the original community were more willing to join the exercise program compared to the seniors who were living in the housing estate (Adj OR = 1.34, 95%CI: 1.01, 1.79). Married older adults were nearly four times more likely to join the program compare to the unmarried older adults (Adj OR = 1.53, 95%CI: 1.18, 1.97). Moreover, working older adults tended to participate in the exercise program compare to the older adults who did not work (Adj OR = 1.47, 95%CI: 1.15, 1.89). Older adults who were in need of walking aids or equipment were almost three times more likely to engage in community group exercise program (Adj OR = 2.92, 95%CI: 2.14, 3.99). Seniors who had secondary caregivers (Adj OR = 1.62, 95%CI: 1.29, 2.03), who experienced a fall in the past six months (Adj OR = 1.53, 95%CI: 1.05, 2.21) tended more to want to join the exercise program. Having underlying diseases, and primary caregivers did not have any significant association with their intention to join a group exercise program. Older adults who exercise, but not regularly, were five times more likely to join the program (Adj OR = 5.10, 95%CI: 3.66, 7.09), and older adults who exercise regularly were almost four times more likely to join, compared to the older adults who did not exercise (Adj OR = 3.94, 95%CI: 2.91, 5.34).

## 4. Discussion

More than half of the study participants were willing to participate in functional training, introduced as a community group exercise. Statistically significant socio-demographic and biological motivators were being of younger age, female, married, and working older persons. We noted that younger elderly participants were more likely to join the exercise program than their counterparts of 75 years and above as expected in other studies among older adult populations [23,24,25]. With increasing age, health and functional ability will decline as well. Therefore, to encourage them to engage in physical activity programs, it is necessary to pay a special attention to the participants’ age-related difficulties and provision of the necessary supportive environment.

### 4.1. Sociocultural Factors

Our finding that higher willingness to participate in a group exercise program among Thai female elderly participants—1.69 times more than their male counterparts—was consistent with other Thai studies, but different from population-based studies in other countries where men preferred outdoor activities [26,27]. In the Thai culture, women perform household chores like cooking, cleaning the house, and gardening more so than men, and this nature of increased physical activity among old women may be an explanation for their intention to join it. Married participants were 1.53 times more willing to join than their single, widowed, or divorced counterparts. Peer support and the influence of emotional support from spouses on their subjective wellbeing may also increase their willingness to join the exercise program [28,29,30]. Receiving social support provided by a partner can also have positive impacts and, thus, program strategies to encourage couples to join together will result in better intervention outcomes. Working at an older age might be the indication of a good health state. In Thai families, mostly men are the head or leaders of the households and currently working elder persons are still physically active and therefore, more likely to join exercise than non-working old participants.

### 4.2. Personal and Behavioral Factors

**Figure 2:**
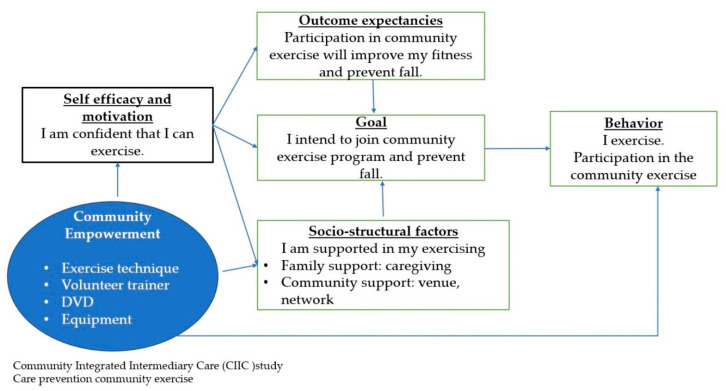
Explanation of Exercise participation, Community Integrated Intermediary Care (CIIC) study: Care prevention community exercise 2019 Note: Care prevention exercise video and technique will be delivered upon request to the corresponding author. DVD= Digital Video Disc of Care prevention exercise.

Regarding personal and behavioral determinants, exercise habit, history of a fall during the preceding six-month period, and functional impairment (in need of walking aids/equipment) were significant associated factors with their intention to join a community group exercise. We did not find any significant impacts of other determinants like smoking, drinking, underlying chronic diseases, level of activities of daily living, and self-perceived health status on their willingness to join the exercise program. Older adults who exercise but not regularly were five times more likely to join the program and older adults who exercise regularly were almost four times more likely, compared to the older adults who did not exercise. It is commonly understood that it is easier to remain active if one is already in the habit of regular physical activity. Prior sedentary behavior of the senior citizens is one of the reasons why they do not participate in physical activity [31]. This strong positive association between exercise habits and willingness to join the exercise program indicated the importance of health promotion approaches, especially lifestyle and behavioral approaches that help inactive older adults adopt physical activity into their daily life, which could then increase the program uptake subsequently. Although only 10.3% of the study participants had a history of a fall in the preceding six months, which was lower than the overall prevalence of 19.8% among Thai elderly [32], the willingness of these participants to partake in our functional training exercise program was 1.5 times higher than those who did not fall in the last six months. Moreover, it was also noted that willingness to join the exercise program was three times higher among the senior citizens who were in need of walking aids/equipment, compared to their counterparts. This increased motivation to join the exercise program by participants with a history of falling and a need for walking aids might have been the result of several awareness campaigns carried out by our research team that informed the participants about the positive effects of functional training on stability and prevention of fall. Theoretically these factors can be explainable in social cognitive theory for behavior change [33]. We identified the need to target self-efficacy to provide proper exercise specifically designed as safe and proper for older people and to empower participants (Figure 2).

### 4.3. Family and Community Environmental Factors

As for family and community environmental determinants, being a resident of their original community and having secondary caregivers had positive impacts on older adults’ willingness to take part in the group exercise program [34,35]. Seniors residing in an original community were more likely to participate in a group exercise compared to those living in a separate, private housing estate. People living in the original community are staying in the traditional private houses, amongst a mixture of rich and poor households. Their community bonding and environmental social safety may be higher than private housing estate. Private housing estate in this study are houses collectively located in the form of residences situated in gated communities, as a result of urban planning and gentrification. Families living there are generally affluent but social connection within and with surrounding native communities is perhaps weaker than the social ties in the original native communities. Public health and community-based program implementation should have awareness not to overlook such gated communities. Family caregivers as the informal caregivers of older adults are the backbone of a current family-based long-term care for seniors. Perceived social support has been evidenced as it has a positive effect on older adults’ physical activity and quality of life [36]. We noted that 53.2% of the study participants, those who had secondary caregivers, were more likely to join the group exercise compared to those who did not have secondary caregivers. Even though having primary caregivers did not have any significant association with their willingness to join the exercise program, emotional, informal, and instrumental support provided by both primary and secondary caregivers play an important role for their motivation, which should be taken into consideration when designing an intervention to promote the well-being of older adults [37].

Some limitations of this study need to be addressed. Baseline cross-sectional survey results could not determine the causality of motivators to join a community group exercise. Moreover, the study participants were mostly mildly dependent in terms of activities of daily living and their functional status would affect the intention to participate in a group exercise, and generalizability of the findings to those who were less functional would be uncertain. Another one is the relatively good health of the participants we recruited, reflected by six percent having poor/very poor self-perceived health status, and, therefore, our findings may not generalize for older persons with poorer health. Despite these limitations, current study has several strengths, including the fact that it was a community survey of community-dwelling senior citizens in Chiang Mai, where older persons occupied 18.2 percent of the entire population. Furthermore, the finding of increased intention to join a community group exercise program among elderly participants with their residences in an original village community have important implications for further studies in Thailand. It showed Thai culture and a sense of social security in an environment where a mix of rich and poor households were sharing the same community resources. It is notable that elderly people with secondary caregivers were more willing to participate in a group exercise. Although the role of secondary caregivers in elderly people’s decisions to join a health promotion program has not been studied yet, an in-depth understanding of this topic might lead to a brand-new discovery for countries like Thailand, where informal caregivers play an important role in care for seniors, ageing in place.

## 5. Conclusions

The findings from this study highlighted the motivators for the elderly to get access to health promotion activities. When people are informed about healthy choices, they tend to choose them. Encouraging physical activity for the older persons is vital to their well-being and health promotion approaches. The result of this study provided a guide for empowerment approach and promotion of older adults’ health. These can be theoretically and conceptually integrated as well as practically applied in the intervention. We identified that persons with exercise habits were more likely to join the community exercise program. We also identified the intention–behavior gap. To minimize the intention–behavior gap, empowering the community and improving self-efficacy are theory-based and evidenced based approaches. To empower individuals, the factors we identified in this analysis—such as original community residents, history of a fall in the last six months, having secondary caregivers, etc.—would be practically useful. This provides a scientific basic to launch a community-based health promotion with the target of behavioral change. We believe that intervention components, such as introducing exercise techniques to the community, training volunteers to be future trainers of the program, stakeholder involvement in designing the intervention, will secure motivation and active participation. Moreover, a community-based exercise program in this study, enabled through community empowerment and social ties, is required to be sustainable. Our findings would help health policy makers design age friendly environment and effective intervention in a community setting for healthy and active ageing of the Thai community-dwelling elderly population.

## Figures and Tables

**Table 1:** Demographic characteristics of the study participants, Maehia, Chiang Mai, Thailand 2019 (*n* = 1509).

Demographic Characteristics	Willingness to Participate in a Community Group Exercise Program		
	Yes	No	Total
	*n* (%)	*n* (%)	*n* (%)
Early elderly <75 years	629 (54.4)	527 (45.6)	1156 (76.6)
Late elderly ≥75 years	146 (41.4)	207 (58.6)	353 (23.4)
Sex			
Male	271 (47.0)	306 (53.0)	577 (38.2)
Female	504 (54.1)	428 (45.9)	932 (61.8)
Residential type			
Housing estate	126 (43.6)	163 (56.4)	289 (19.2)
Original community	649 (53.2)	571 (46.8)	1220 (80.8)
Marital status			
Married	500 (55.4)	403 (44.6)	903 (59.8)
Not married (single, separated, divorced, widowed)	275 (45.4)	331 (54.6)	606 (40.2)
Education			
No formal education	28 (37.8)	46 (62.2)	74 (4.9)
Primary school completed	481 (56.2)	375 (43.8)	856 (56.7)
Secondary school and above	266 (45.9)	313 (54.1)	579 (38.4)
Still working			
No	499 (47.9)	543 (52.1)	1042 (69.1)
Yes	276 (59.1)	191 (40.9)	467 (30.9)
Exercise habit			
No Exercise	86 (25.7)	249 (74.3)	335 (22.2)
Exercise but not regularly	410 (55.9)	324 (44.1)	734 (48.6)
Exercise regularly	279 (63.4)	161 (36.6)	440 (29.2)
Current Smoking			
No	726 (51.5)	685 (48.5)	1411 (93.5)
Yes	49 (50.0)	49 (50.0)	98 (6.5)
Current Alcohol drinking			
No	619 (50.9)	596 (49.1)	1215 (80.5)
Yes	156 (53.1)	138 (46.9)	294 (19.5)
Living arrangement			
Stay alone	96 (48.7)	101 (51.3)	197 (13.1)
Stay with Spouse	303 (56.5)	233 (43.5)	536 (35.5)
Son/Daughter/(son or daughter in law)	302 (49.3)	311 (50.7)	613 (40.6)
Others (relatives, grandchildren)	50 (52.6)	45 (47.4)	95 (6.3)
Siblings	24 (35.3)	44 (64.7)	68 (4.5)
Have primary caregiver			
No	74 (49.7)	75 (50.3)	149 (9.9)
Yes	701 (51.5)	659 (48.5)	1360 (90.1)
Have secondary caregiver			
No	322 (45.6)	384 (54.4)	706 (46.8)
Yes	453 (56.4)	350 (43.6)	803 (53.2)
Underlying diseases			
No	301 (53.8)	258 (46.2)	559 (37.0)
Yes	474 (49.9)	476 (50.1)	950 (63.0)
Activities of daily living (ADL) by Barthel’s ADL total scores			
Mild Dependent ≥12	770 (52.2)	705 (47.8)	1475 (97.7)
Moderate to Severe Dependent <12	5 (14.7)	29 (85.3)	34 (2.3)
Perceived health status			
Poor/very poor	33 (36.3)	58 (63.7)	91 (6.0)
Neutral	388 (56.2)	303 (43.8)	691 (45.8)
Good/very good	354 (48.7)	373 (51.3)	727 (48.2)
Experienced fall last 6 months			
No	685 (50.6)	668 (49.4)	1353 (83.7)
Yes	90 (57.7)	66 (42.3)	156 (10.3)
Needs for walking aids/equipment			
No	594 (47.8)	649 (52.2)	1243 (82.4)
Yes	181 (68.0)	85 (32.0)	266 (17.6)

**Table 2:** Factors affecting the willingness of community dwelling elderly persons for participation in a community group exercise program, Maehia, Chiang Mai, Thailand 2019.

Demography	Willing to Participate in a Community Group Exercise Program			
	Frequency (%)	Adjusted OR	95% Confidence Interval	
			Lower	Upper
Age				
≥75 years	146 (41.4)	Referent		
<75 years	629 (54.4)	1.39 *	1.05	1.85
Gender				
Male	271 (47.0)	Referent		
Female	504 (54.1)	1.69 **	1.33	2.15
Residential type				
Housing estate	126 (43.6)	Referent		
Original community	649 (53.2)	1.34 *	1.01	1.79
Marital status				
Not married (single, divorced, separated, widow)	275 (45.4)	Referent		
Married	500 (55.4)	1.53 *	1.18	1.97
Underlying disease				
No	301 (53.8)	Referent		
Yes	474 (49.9)	0.80	0.64	1.01
Elderly is still working				
No	499 (47.9)	Referent		
Yes	276 (59.1)	1.47 *	1.15	1.89
Needs for walking aids/equipment				
No	594 (47.8)	Referent		
Yes	181 (68.0)	2.92 **	2.14	3.99
Exercise habit				
No Exercise	86 (25.7)	Referent		
Exercise but not regular	410 (55.9)	5.10 **	3.66	7.09
Regular exercise	279 (63.4)	3.94 **	2.91	5.34
Experienced fall last 6 months				
No	685 (50.6)	Referent		
Yes	90 (57.7)	1.53 *	1.05	2.21
Have primary caregiver				
No	701 (51.5)	Referent		
Yes	74 (49.7)	0.80	0.54	1.19
Have secondary caregiver				
No	322 (45.6)	Referent		
Yes	453 (56.4)	1.62 **	1.29	2.03

* *p* value < 0.05, ** *p* value < 0.01, Adjusted OR = Adjusted odd ratio.

## Data Availability

The data presented in this study are available on request from the corresponding author. The data are not publicly available because this study was a sub-group analysis of baseline data of an intervention clusters from a cluster randomized trial and the final analysis was ongoing and publications of the whole cluster randomized trial has not finished yet.
